# Thought habits and processing modes among Japanese university students do not influence dynamic associations between rumination and negative affect

**DOI:** 10.1038/s41598-024-55174-7

**Published:** 2024-03-19

**Authors:** Kohei Kambara, Shushi Namba, Satoshi Yokoyama, Akiko Ogata

**Affiliations:** 1 Faculty of Psychology, Doshisha University, Kyo-Tanabe-Shi, Kyoto Japan; 2 Graduate School of Humanities and Social Sciences, Hiroshima University, Higashi-Hiroshim-Shi, Hiroshima Japan; 3 Faculty of Humanities, Niigata University, Niigata-Shi, Niigata Japan

**Keywords:** Psychology, Depression

## Abstract

The integrated model of rumination argues that two trait factors—negative thinking habits and processing modes—get people stuck in maladaptive rumination. There is little evidence showing whether these factors influence the daily dynamic associations between rumination and negative moods. To address this, in this study, we conducted an experience-sampling method on Japanese university students. We recruited 92 Japanese university students and assessed their daily rumination and negative affect (NA) eight times a day for seven days. We examined the effects of habits and processing modes on the dynamic associations between rumination and negative moods using dynamic structural equation modeling. We found that individuals were more likely to ruminate when they experienced NA. However, contrary to previous findings, this study’s participants did not experience NA after engaging in rumination. Moreover, we did not detect any significant trait factor effect on these dynamic associations. Our findings imply that individuals are more likely to engage in rumination after experiencing NA, but the reverse association, particularly the autoregression of rumination, may not be maintained in natural daily life. Furthermore, negative thinking habits and processing modes may not influence the daily dynamic associations between rumination and NA among Japanese university students.

## Introduction

When people inadvertently miss a work assignment and have the misfortune of angering their bosses, they might engage in one thought, such as “What a stupid person I am,” for a while. However, if the thought keeps coming up beyond their control and they have trouble stopping it, the thought may be classified as rumination. State rumination comprises excessive, obsessive, and repetitive negative thoughts, characterized by recurrent, uncontrollable, and evaluative thinking about one’s feelings, personal concerns, and negative events^1^. Many studies have shown that individuals with a high tendency to ruminate are more likely to exacerbate their depressive symptoms^2,3^. Individuals engaging in state rumination evoke negative moods during subsequent hours^4–6^. Although rumination is a maladaptive thought that causes unconstructive consequences, it is difficult for individuals who often experience rumination to stop that thinking^7^.

To explain this, Watkins and Roberts^1^ provided an integrated rumination model called the HExAGoN model, which indicates that the habits of negative thinking and processing modes are elementary factors that maintain rumination. The model includes other proximal factors that maintain maladaptive rumination, such as executive functions, goal discrepancies, and negative bias^1^. In this study, we focus on the habits and processing modes aspects because the clinical approach for rumination often addresses these two factors^7^. Individuals who struggle with state rumination have often learned negative thinking as a habitual characteristic^8^. As habits are formed through stimulation (mental contexts) and response styles (triggered through action associations learned through prior performances), which are strongly connected, individuals have the tolerance to eliminate or reverse them^9^. Individuals with negative thinking habits are more likely to use ruminative thinking after experiencing negative moods^6,10^. Processing modes are thought mindsets that are classified into two styles: Abstract—evaluative and analytical thinking, focusing on the implications and consequences of events and feelings; and Concrete—experiential and contextual thinking, focusing on the details of events and feelings^11^. Individuals who tend to engage in rumination usually adopt high abstract and low concrete processing modes^12,13^. Therefore, a biased processing mode is often found among individuals who engage in rumination. This biased processing mode causes negative consequences. For instance, an experimental study demonstrated an exacerbation of negative moods among participants who were induced into the abstract processing mode. However, the modification of these moods was observed among those who were induced into the concrete processing mode^14^.

From the perspective of the HExAGoN model, as mentioned in the paragraph above, a bidirectional relationship can be considered between state rumination and negative affect (NA). When considering the pathway from state rumination to NA, the rumination is thought to amplify NA arising from stressful events owing to its tendency to have more abstract processing modes and fewer concrete processing modes^12–14^. Indeed, simply inducing state rumination increases negative moods compared to distraction inducement^15^. Therefore, rumination can be considered a factor that increases NA. However, when considering the influence of NA on state rumination, because state rumination has a habitual nature that automatically occurs in response to specific contextual stimuli, such as NA, it is believed that NA increases the time and frequency individuals spend ruminating^6,9^. Thus, NA can also be considered a factor that increases state rumination. In summary, a bidirectional relationship is considered between state rumination and negative moods.

Recent research has examined this bidirectional relationship. Developments in technology enable researchers to measure state rumination using intensive longitudinal data, which includes several timepoints during intervals, such as a day, week, and so on^16^. For instance, the experience-sampling method (ESM), a technique for measuring state rumination that is usually conducted by asking participants about their state rumination and mood states during a day and involves continuous measurements daily for a week^5^, has the merit of reducing the recall bias of emotional variables^17^. Previous research that used the ESM to examine the daily associations between state rumination and NA found that state rumination exacerbates NA among individuals^18,19^. Individuals are more likely to engage in state rumination if they encounter stressors^20^, which might be amplified NA. In summary, previous ESM studies found that state rumination predicted NA, and negative moods could predict state rumination.

For intensive longitudinal data, recent research has recommended using dynamic structural equation modeling (DSEM)^21^, an integrated statistical approach that can simultaneously analyze differences between multi-level properties and time-series features^21^. Thus, DSEM provides some insights on how different individuals change their rumination through their negative moods based on time-series trajectories. A few studies have examined the dynamic associations between state rumination and NA using DSEM^6,22^. For instance, Blanke et al.^22^ conducted DSEM on prior research data collected from university students via the ESM using different datasets. In Study 1, they analyzed data on state rumination and negative moods of participants who answered ten times a day for seven consecutive days, whereas in Study 2, they analyzed the data of participants who answered six times a day for nine consecutive days. The results showed that individuals were more likely to feel negative moods when they previously engaged in state rumination, and vice versa. Moreover, to the best of our knowledge, the only challenge faced by Hjartarson et al.^6^ was revealing the effects of the habit of negative thinking on the dynamic relationship between state rumination and negative moods, which they measured ten times a day for six consecutive days. They found that the habit of negative thinking predicts an increase in the cross-lagged effects from NA to state rumination.

Although previous studies that examined the dynamic associations between state rumination and negative moods have implied that negative thinking habits influence these dynamic associations, little attention has been paid to the effects of processing modes, which are also considered factors that maintain rumination^1,8^. The integrated HExAGoN model indicates some risk factors relating to individuals’ likelihood of maintaining their maladaptive rumination. However, ESM studies could overlook the perspective of multiple aspects of these risk factors. Therefore, this study shed light on the way in which multiple factors, processing modes, and habits influence the dynamic associations between rumination and NA simultaneously. This study might provide new insight into the mechanism of the way in which rumination increases NA, and vice visa. Many experimental studies have implied that processing modes amplify negative moods^14,23–27^. Exploring the ways in which processing modes influence daily dynamic associations between rumination and negative moods beyond experiment laboratories would help us further understand the mechanism behind the ways in which processing modes maintain rumination. Given previous laboratory findings, we predict that the concrete processing mode will decrease the coefficients of autoregression in state rumination as it promotes adaptive behaviors^27–29^. In contrast, we consider that the abstract processing mode, which exacerbates negative moods following harmful incidents, such as a failure, may make it easier for individuals to engage in state rumination after experiencing negativity^14^.

Our aim is to reveal the ways in which the multiple factors that maintain rumination influence the dynamic associations between state rumination and NA. Therefore, we considered two factors: habits of negative thinking and processing modes. Although the processing mode was viewed as a state variable in previous studies^30^ or manipulated in experiments^14^, this study treated the processing mode as a trait factor because this mode demonstrates relative stability within individuals. Indeed, previous studies using longitudinal surveys have shown that processing modes are significantly associated with depression in between-person levels^31^. We shall adopt the ESM approach to collect intensive longitudinal data from Japanese university students and analyze it via DSEM. We considered the following hypotheses. The first and second hypotheses were related to the replication of previous findings, which were established through the ESM, and the third hypothesis was related to this study’s original examination. Hypothesis 1—Individuals reported engaging in more rumination when they felt negative moods, and vice versa, which has been shown in previous studies^6,22^; Hypothesis 2—The habit of negative thinking amplifies dynamic associations, especially state rumination, which is triggered more by NA^6^; and Hypothesis 3—Individuals with high abstract and low concrete processing modes are more likely to exacerbate negative moods when they engage in rumination.

## Results

The ESM received 3594 answers, whose mean was 39.49 (70.03%). After exclusions owing to insufficient adherence if participants completed ESM answers, with less than 50% referring to previous ESM study criteria^22^ or consistently providing the same response number for all ESM signals, which raises concerns about the sincerity of their responses in the investigation, there were a total of 3,382 answers, whose mean was 41.75 (74.55%). The descriptive statistics and correlations are listed in Table 1. The mean of depressive symptoms measured using the Center for Epidemiologic Studies Depression Scale (CES-D) was 16.96. Although the original paper of CES-D argued that 16 points is the cutoff score of clinical depressive symptoms^32,33^, the cutoff has been indicated as too low to capture clinical depressive symptoms^34,35^. Indeed, previous studies that included Japanese university students showed CES-D scores similar to those observed in this study^31,36^. Thus, the depressive symptoms of the current sample population may not be viewed as clinical level symptoms.

**Table 1:** Descriptive statistics.

		*Mean*	SD	1	2	3	4	5	6
Trait measures									
1	Habit of negative thoughts	49.62	16.86						
2	Abstract processing mode	23.00	5.10	.69**					
3	Concrete processing mode	17.85	3.54	− .37**	− .22**				
Covariate measures									
4	Depression	17.13	6.18	.42**	.45**	− .15**			
5	Rumination	49.61	10.41	.61**	.64**	− .13**	.43**		
ESM measures									
6	State rumination	5.23	3.49	.18**	.14**	− .09**	.20**	.10**	
7	Negative affect	4.85	2.50	.12**	.12**	− .08**	.25**	.10**	.65**

**p < .01, *p < .05.

*ESM* experience-sampling method, *SD* standardized deviation. The ESM measures mean daily responses about state rumination and negative affect.

### Model comparisons to identify the primary model

First, we conducted a model comparison using the deviance information criterion (DIC) index. To decide upon the model using DSEM, we compared the following three models: Model A, which included only state variables; Model B, in which the effects of habits and processing modes were added to Model A; and Model C, in which covariates, such as depression, gender, and age were added to Model B. Each model’s DIC index and coefficients of dynamic relationships are described in Table 2. The results indicate that model C had a better DIC index compared to models A and B. There were no differences in the tendency of significance in the autoregressions and cross-lagged effects among the models. Given these results, we applied Model C as the main model, rather than Models A and B.

**Table 2:** The results of autoregressions and cross-lagged effects in each model by DSEMs.

			*b*	*SE*	β	95% CI low	95% CI high
Model A	DIC	115,155.82					
Autoregression							
State rumination	On	State rumination	0.08	0.06	0.17	− 0.09	0.43
Negative affect	On	Negative affect	0.30	0.06	0.69	0.04	1.03
Cross-lagged effects							
State rumination	On	Negative affect	− 0.03	0.04	− 0.11	− 0.38	0.16
Negative affect	On	State rumination	0.29	0.10	0.37	0.11	0.64
Model B	DIC	− 161,043.08					
Autoregression							
State rumination	On	State rumination	0.09	0.06	0.18	− 0.07	0.43
Negative affect	On	Negative affect	0.30	0.06	0.70	0.40	1.04
Cross-lagged effects							
State rumination	On	Negative affect	− 0.03	0.04	− 0.08	− 0.35	0.18
Negative affect	On	State rumination	0.30	0.10	0.37	0.12	0.65
Model C	DIC	− 448,048.31					
Autoregression							
State rumination	On	State rumination	0.07	0.07	0.14	− 0.13	0.42
Negative affect	On	Negative affect	0.31	0.06	0.69	0.39	1.02
Cross-lagged effects							
State rumination	On	Negative affect	− 0.05	0.05	− 0.14	− 0.41	0.14
Negative affect	On	State rumination	0.28	0.12	0.34	0.05	0.63

*DIC* deviance information criterion, *SD* standardized deviation. The state rumination and negative affect were measured using the experience-sampling method.

### How habits and processing modes influence dynamic relationships between daily rumination and NA

**Figure 1:**
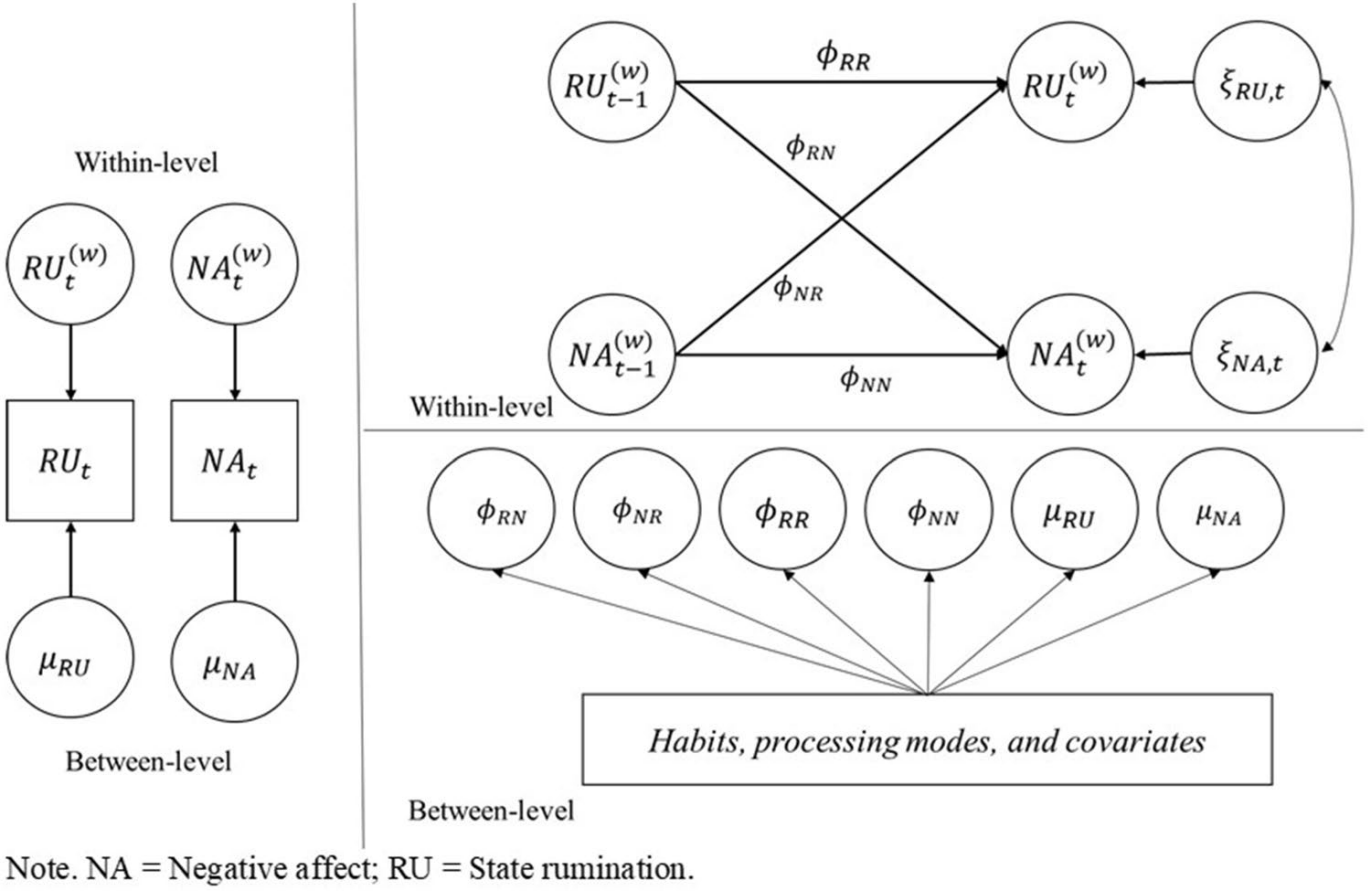
The present model is used in this study as Model C. The left side panel represents decomposing of ESM variables into within-person and between-person level components. The right and upper side panels show the dynamic associations at within-person levels. φRR and φNN represent autoregression effects, and φRN and φNR represent cross-lagged effects. The right and lower side panels describe the regression models at between-person levels. Thus, habits, processing modes, and covariates were included in multiple regressions as predictive variables, and each coefficient for within-person and between-person levels was included as a dependent variable.

To reveal the dynamic associations between state rumination and NA and examine the influence that habits and processing modes had on them, we conducted two-level first-order vector autoregressive models (Fig. 1).

The results at the within-person level are listed in Table 2, where significant autoregressions among NA (*b* = 0.31, *SD* = 0.06, β = 0.69, CI 95% [0.39: 1.02]) are found. However, the model indicated that there were no significant autoregressions in state ruminations (*b* = 0.07, *SD* = 0.07, β = 0.14, CI 95% [− 0.13: 0.42]). Thus, the autoregressive association existed only in the NA. To observe the cross-lagged effects, we can find significant effects of NA on state rumination (*b* = 0.28, *SD* = 0.12, β = 0.34, CI 95% [0.05: 0.63]), although the reverse effect was not detected (*b* = − 0.05, *SD* = 0.05, β = − 0.14, CI 95% [− 0.41: 0.14]).

The results at the between-person level can be observed in Table 3. Surprisingly, habits and processing modes had no significant influence on autoregressions and cross-lagged effects. In contrast, depressive symptoms increased the autoregression of daily NA (*b* = − 0.03, *SD* = 0.01, β = 0.41, CI 95% [0.14: 0.66]). Thus, individuals with high depressive symptoms are likely to amplify their negative moods after they feel NA.

**Table 3:** The effects of habits, processing modes, and covariate variables on dynamic associations in Dynamic Structural Equation Modeling based on Model C.

	*b*		*b*	*SD*	*β*	CI low	CI high	*b*	*SD*	*β*	Cl low	CI high	*b*	*SD*	*β*	CI low	CI high
Variables			HINT					AAT					CET				
Means																	
State rumination			0.03		0.22	- 0.10	0.53	0.00	0.08	− .0.01	0.31	− .0.32	0.01	− .0.09	− .0.01	0.22	0.09
Negative affect			0.01		0.06	- 0.26	0.37	0.02	0.06	− .0.07	0.25	− .0.37	0.01	− .0.06	− .0.01	0.22	0.06
Autoregression																	
State rumination	On	State rumination	− .0.01	0.01	− .0.24	− .0.60	0.14	0.02	0.02	0.16	− .0.23	0.54	− .0.04	0.02	− .0.26	− .0.52	0.03
Negative affect	On	Negative affect	0.01	0.01	0.20	− .0.18	0.57	− .0.01	0.02	− .0.18	− .0.53	0.19	0.03	0.02	0.22	− .0.09	0.49
Cross-lagged path																	
State rumination	On	Negative affect	0.00	0.00	− .0.13	− .0.50	0.26	0.00	0.01	0.07	− .0.34	0.47	− .0.02	0.01	− .0.27	− .0.54	0.02
Negative affect	On	State rumination	0.01	0.01	0.28	− .0.10	0.64	− .0.04	0.03	− .0.24	− .0.61	0.14	0.02	0.03	0.07	− .0.22	0.34

Variables			DEP					AGE					GENDER				
Means																	
State rumination			0.06	0.05	0.16	- 0.10	0.40	0.34	0.16	0.24	0.02	0.46	1.03	0.66	0.18	0.40	0.66
Negative affect			0.08	0.04	0.26	0.01	0.49	0.28	0.11	0.28	0.05	0.49	0.66	0.46	0.16		
Autoregression																	
State rumination	On	State rumination	− .0.01	0.01	− .0.15	− .0.42	0.15	0.03	0.04	0.12	− .0.14	0.37	0.07	0.15	0.06	− .0.20	0.31
Negative affect	On	Negative affect	0.03	0.01	0.41	0.14	0.66	− .0.03	0.03	− .0.11	− .0.35	0.14	− .0.10	0.13	− .0.10	− .0.36	0.17
Cross-lagged path																	
State rumination	On	Negative affect	− .0.01	0.01	− .0.27	− .0.54	0.03	0.02	0.02	0.12	− .0.15	0.39	0.10	0.10	0.14	− .0.13	0.40
Negative affect	On	State rumination	0.03	0.02	0.23	− .0.06	0.49	− .0.04	0.06	− .0.08	− .0.33	0.17	0.02	0.24	0.01	− .0.25	0.26

*AAT* abstract processing mode, *CET* concrete processing mode, *DEP* depressive symptoms, *HINT* habit of negative thoughts.

### Sensitivity analysis

Our exclusion owing to low compliance to answer each signal based on previous criteria^22^ may cause bias in the current results. Therefore, we conducted a sensitivity analysis. We conducted DSEM using Model C, which is our primary model, including all participants who entered this study. The result showed that there was no difference in the pattern of results, except for the effects of depressive symptoms on the cross-lagged path from state rumination to NA (*b* = − 0.02, *SD* = 0.01, β = − 0.30, CI 95% [− 0.56: − 0.01]). Thus, the primary result was almost replicated, which shows robustness of the findings. The details of the result can be accessed in the Open Science Framework (https://osf.io/h348t/?view_only=8bdfedd3b71f4341a135df7383e1b227).

## Discussion

This study aimed to examine whether habits of negative thinking and processing modes, which are considered factors maintaining maladaptive rumination in the integrated rumination model called the HExAGoN model, influence daily dynamic associations between rumination and NA. Although the results showed autoregression and a cross-lagged effect of NA on state rumination, autoregression and a cross-lagged effect of state rumination on NA, which were found in a previous study^6,22^, were nonexistent. Moreover, habits and processing modes could not predict the exacerbation of rumination autoregression or cross-lagged effects. Therefore, our hypotheses were not validated by our results, and we could not replicate the results of a previous study using DSEM in a Japanese university students’ sample. These issues are discussed in the following section.

As this study showed no autoregression in rumination, its results were inconsistent with those of previous studies. This study, which used the ESM, set eight time signals on a specific day and asked participants the degree of their rumination during the last fifteen minutes. In comparison, a prior study that showed significant autoregression of rumination had measured rumination ten times per day by asking participants about their degree of engaging in rumination at the present moment in each of the ten time signals. The difference implies that because the sample was not a clinical group (subclinical depressive symptoms or major depressive disorders), they could not maintain their rumination for long spans of time. Therefore, our eight ESM time signals could not capture their continuous rumination. Indeed, a previous study reported that university students’ mean of engaging in rumination was 26.2 min (*SD* = 81.8 min)^37^. Given this, our research could represent natural state rumination among university students, and most of them may be unlikely to retain their negative thoughts. Future research may need to measure state rumination more continuously, for instance, by conducting the ESM at 30-min intervals, or by focusing on highly ruminative individuals who may engage in rumination for over an hour.

We hypothesized that habits of negative thinking amplified the cross-lagged effects of state rumination on NA, and the abstract processing mode increased the intensity of cross-lagged effects of state rumination on NA. Previous studies have implied that habits of negative thinking are one of the trait factors through which individuals can easily start ruminating and get stuck in maladaptive thoughts^1,8,9^. A study in which rumination was captured using the ESM showed that such habit tendencies exacerbate the dynamic associations, and NA can cause subsequent ruminative thinking^6^. Moreover, we predicted the effect of processing modes on these associations based on previous experimental studies^14,23–27^ and theory^1^. However, this study does not support these associations and our hypotheses. Habits of negative thinking represent a degree of intrusion and difficulty in controlling momentary negative thoughts. In this study, the cross-lag is spaced by more than 60 min, indicating a relationship that extends beyond the immediate moment to some extent. Therefore, the habits may not have strengthened the relationship where negative feelings at a specific point predict subsequent state rumination. Moreover, in previous studies, the abstract processing mode amplified negative moods through experiences of failure^14,23–27^. In this study, we did not assess the contexts of NA, which might uncover the effects of processing modes. Further examination is required to reveal the effect of habits and processing modes on the dynamic associations between state rumination and negative moods. For instance, in future studies, it may be helpful to clarify the effect of manipulating processing modes and investigate their influence using the ESM.

Our findings have several implications. Our ESM and DSEM approaches imply that natural rumination and mood associations among university students may not be influenced by integrated factors maintaining rumination, as explained by the HExAGoN model. Given our findings and evidence from previous studies, it is implied that processing modes may exacerbate dynamic associations between rumination and moods in a clinical setting, but not among non-depressed individuals. For instance, interventions for bias of processing modes contribute to improving depressive symptoms in clinical settings^38^. However, further studies that examine the effects of processing modes on these dynamic associations among clinical individuals, such as those having excessive rumination or dysphoric moods, are needed. Nevertheless, this study reveals the ways in which multiple factors of the HExAGoN model influence natural state rumination and moods among university students beyond experimental settings.

Moreover, the findings of this study suggest the potential for expanding the HExAGoN model. The model assumes a hypothetical bi-directional association between processing modes and the habits of negative thinking^1^. The abstract processing style amplifies perceptions of goal discrepancies as this mode leads to poor problem solving in difficult situations^39^. However, this assumption has not been empirically demonstrated. The results of this study that processing modes do not increase the cross-lagged association between state rumination and NA do not support the idea that processing modes influence developing habits of rumination. Further investigation into this relationship is warranted. For example, examining the simultaneous measurement of changes in daily processing modes, mood fluctuations, and sate rumination through methods, such as the ESM, and conducting analyses similar to DSEM employed in this study, may provide new insights. This could shed light on the intricate dynamic association between processing modes, mood, and state rumination.

Although direct comparisons must be approached with caution, there is an intriguing aspect from the perspective of cultural differences. Our sample was characterized as a Japanese culture population. One cross-cultural questionnaire survey involving European Americans and East Asians showed that cultural differences influencing the effect of rumination on increasing depressive symptoms were caused by a self-doubt attribution style^40^. This difference may suggest that Japanese individuals often report higher rumination levels but show fewer depressive symptoms, which might also explain why the study did not find cross-lagged effects of state rumination on NA. Nevertheless, we cannot conclude or argue strongly about the differences between this study’s results and those of the previous survey using DSEM as we did not compare other cultural contexts.

In practice, our study used DSEM for intensive longitudinal data, and the results imply that this approach can provide various data perspectives rather than the previous conventional multilevel modeling^41^. The analysis can capture dynamic fluctuations, such as autoregression and cross-lagged effects, which are close to our complex internal activities of daily life. Moreover, these approaches can inhibit statistical bias when using intensive longitudinal data^21^. The ESM approach is helpful for examining the intrinsic associations between thoughts and moods^17^.

This study has several limitations. First, its sample comprised only Japanese university students. The distribution of state rumination and NA generally leans in the direction of scoring less. This tendency is consistent with previous ESM studies on non-depressed participants^6,19^. One study reported that individuals with depressive symptoms were more likely to show fluctuations between rumination and moods^5^. Although it is not clear whether the lack of fluctuations influenced this study, DSEM could not compute the standardized coefficient of autoregressions of NA as problems occurred in several iterations owing to autoregression coefficients of NA greater than 1. An ESM study to explore the differences between clinical or analog samples may provide further insight into the ways in which proximal factors influence dynamic associations. Second, missing data may have influenced this study’s estimation. The ESM approach requires careful collection to reduce the missing rate, although this approach is useful for examining the daily dynamic associations between thoughts and moods^17^. Our average completion rate was approximately 70%, which is not less than that of previous ESM research^6^. However, the missing rate implies that our analyses could not capture 30% of the variance. To address the missing rate, we informed participants that they could obtain greater incentives if they endured a high complication rate. Nevertheless, there will be human error, wherein participants inevitably forget to answer. Therefore, to increase the collection rate, it is necessary to devise ways, such as wearable devices and automated collection. Finally, this study has several methodological limitations: the number of signals in the ESM, the issue of missing data, and the measurement validity of state rumination. The correlation coefficient between RRS and state rumination in this study was statistically significant but relatively low (*r* = 0.10), and that between state rumination and NA was high (*r* = 0.65). The differences in measurement items between the current study and previous studies may have influenced the results. Specifically, given that we argue about cultural differences in rumination, future research may need to tailor measurement methods to each cultural context for more nuanced insights. Essentially, it is crucial to enhance the methodological validity before attempting to replicate results similar to those of previous studies. If there is a desire to deepen the understanding of the relationship between habits and processing modes, it is essential to conduct a replication study to validate the results of this study.

Our findings suggest two noteworthy points. First, the daily dynamic autoregression of state rumination among Japanese university students might be shorter than that of previous findings of ESM studies. Second, processing modes may not influence daily rumination among general university students. Rather, these modes may be strongly associated with clinical-level rumination. However, further research is required to examine the influence of these factors on dynamic associations by manipulating habits and processing modes. Finally, it is worth nothing that our findings have a reproducibility issue. The issue lies not only in the lack of existing theoretical contributions but also in the insufficient accumulation of empirical data. A multinational replication comparison based on the methodology of this study, including our hypotheses related to cultural backgrounds, is crucial. This would contribute to a meaningful validation of the resistance theory and propel the examination to the next stage.

## Methods

### Participants

We selected participants via a system that advertised participation in psychological experiments at the university, recruiting 92 Japanese university students who participated in a study titled “Investigation of the relationship between thoughts and moods in daily life.” The determination of the sample size in this study was referred from previous ESM study designs^6,22^. Although we had no exclusion criteria, the participants needed to have their smartphones to enroll in the study. We did not assess their clinical state using any structural interviews. The inclusion criterion was that they had to be university students. Participants’ mean age was 21.14 years (standard deviation (*SD*) = 1.70 years), and 68 were female. After the investigation, 11 participants who had completed 50% or less ESM questions were excluded owing to inadequate compliance in line with previous ESM study criteria^22^. The final sample comprised 81 participants, with a mean age of 21.20 years (*SD* = 1.78 years), of which 61 were female.

### Procedures

**Figure 2:**
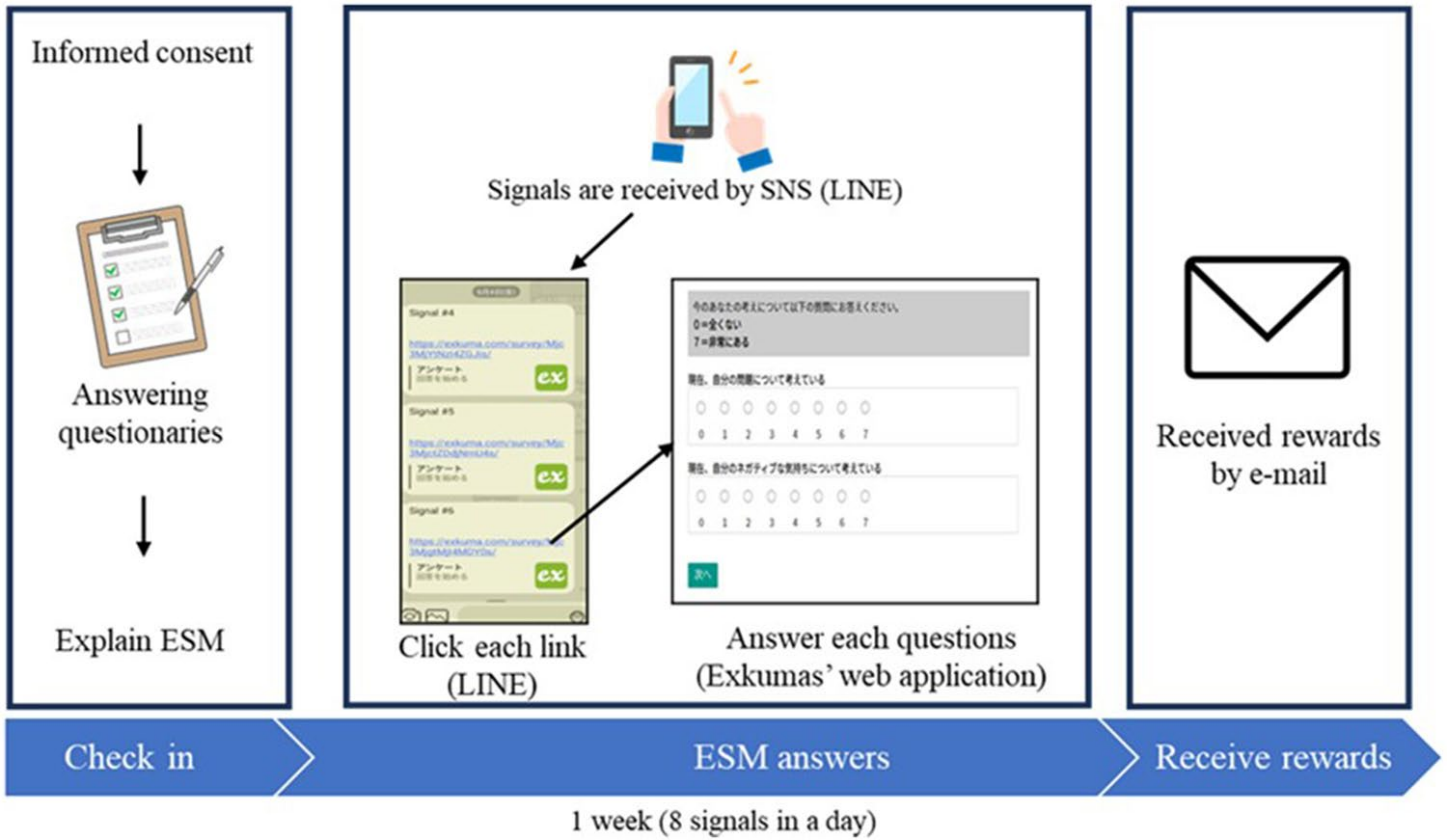
The procedures of the current study. First, the participants checked in at the laboratory. Afterwards, they received experience-sampling method (ESM) signals eight times a day using the SNS application (LINE) and web application (Exkuma). When they completed the ESM signals, they received rewards for participating in the study via e-mail.

This study’s plan and implementation were based on ethical considerations in accordance with the Declaration of Helsinki, and it commenced after receiving approval from the ethical committee of the Graduate School of Education, Hiroshima University. The flow of this procedure is described in Fig. 2. In the laboratory session, participants answered questions about their trait measures, and how to answer the ESM signals was explained to them for approximately 20 min. First, participants were informed about the study’s purpose, what needed to be done, and the incentives for participation, and they agreed to participate by providing written consent. After providing informed consent, they answered questionnaires that measured habits of negative thinking, abstract processing styles, maladaptive rumination, and depressive symptoms. Subsequently, participants were registered in the ESM application (Exkuma, https://exkuma.com/), which could send ESM signals via the social networking service (SNS) application (LINE: https://line.me/ja/), and they received an explanation on how they should answer these signals. The next day onwards, the participants received eight ESM signals via a smartphone SNS application daily, for seven consecutive days over a 14-h period (9 a.m. to 11 p.m.). Each signal was semi-randomized and sent following a prior signal at a minimum interval of 60 min in a day. Participants answered questions about their state rumination and NA when they noticed the signals. However, if they missed the signals for more than 15 min, the ESM application restricted their answering. After completing the ESM periods, the participants were debriefed, and they received an incentive to participate via e-mail that sloped according to their percentage of responding. They were informed that they would receive a maximum of 3,000 Japanese yen (approximately 25 US$) if they completed more than 70% of the signals.

#### Questionnaires’ measures

##### Processing modes: Mini-Cambridge Exeter Repetitive Thought Scale (CERTS)

This scale was originally developed by Douilliez et al.^12^, and its Japanese version was developed, revised, and published by Kambara et al.^42,43^. It measures two dimensions of processing modes: Abstract (item example: “My thinking tends to get stuck in a rut, involving only a few themes”) and Concrete (item example: “I can grasp and respond to changes in the world around me without having to analyze the details”). The scale’s factor structure, internal consistency, and criteria validity have been confirmed in previous studies^43^. Each item was rated on a 4-point scale ranging from 1 (“almost never”) to 4 (“always”). The subscale scores ranged from 6 to 24. High scores on both subscales indicated a high tendency to engage in each type of thinking.

##### Habit of negative thinking: Habit Index of Negative Thought (HINT)

This scale was developed by Verplanken et al.^9^, but it had no Japanese version. Therefore, to develop this, we conducted a preliminary survey. First, we contacted its original author and obtained approval for the translation. Next, we independently translated its items into Japanese and integrated the two drafts. Thereafter, we conducted back-translations via an English proofing service (Editage: https://www.editage.jp/) and sent it to the original author to confirm whether each item was consistent with the original items. Through confirmation, we developed its Japanese version. Second, we conducted a survey among 500 Japanese adults to examine the scale’s reliability and validity. Finally, we found that the Japanese version of the HINT showed good reliability and a similar correlation with the original HINT. The results are described in the Open Science Framework (https://osf.io/h348t/?view_only=8bdfedd3b71f4341a135df7383e1b227). This questionnaire comprises 12 items that require complying with instructions and that ask participants about their degree of habits relating to negative thoughts (item example: “Thinking negatively about myself is something… I frequently do”). The participants responded to items on an 8-point scale ranging from 0 (“strongly disagree”) to 7 (“strongly agree”).

##### Depressive symptoms: Center for Epidemiologic Studies Depression Scale (CES-D)

We assessed depression using the Japanese version of the CES-D^32,33^. We used depressive symptoms as a covariate because depression influences the occurrence probability of sadness. The CES-D comprises 20 items that assess depressive symptoms (item example: “I was bothered by things that usually don’t bother me”). Participants answered each item in terms of how frequently they had experienced a particular symptom in the last seven days. Response options ranged from 0 (“rarely or none of the time; less than one day”) to 3 (“most or all of the time; 5–7 days”). The total scores ranged from 0 to 60, with higher scores indicating more depressive symptoms. This scale has demonstrated good validity for assessing depression^36^.

##### Rumination tendency: Ruminative Responses Scale (RRS)

We assessed maladaptive rumination using the RRS^44^ for confirmation of the criteria validity of the Japanese version of the HINT scale, which was used, as it demonstrated acceptable reliability and validity^45^. This questionnaire comprises 22 items that assess the frequency of ruminative thoughts related to feelings of depression or sadness. The participants responded to items on a 4-point scale ranging from 1 (“almost never”) to 4 (“almost always”).

#### ESM measures

##### State rumination

We used two items to assess the degree of state rumination in each signal, referring to previous ESM studies^46,47^. Participants answered the extent to which they engaged in rumination at a given moment on a scale ranging from 0 (“not at all”) to 7 (“very much”) as follows: “At the moment, I am thinking about my problems” and “At the moment, I am thinking about my negative feelings.” In this study, the rumination scale had an α = 0.82, which was small but significantly correlated with RRS (*r* = 0.11, *p* < 0.01).

***NA.*** We measured participants’ current moods using three items on the Positive and Negative Affect Scale^48^. We selected the scale’s items based on a previous ESM study^6^. NA consisted of three items: “I feel sad right now,” “I feel irritable right now,” and “I feel guilty right now.” In this study, the NA had an α = 0.74, and was moderately correlated with CES-D (*r* = 0.24, *p* < 0.01).

### Statistical analyses

We tested our hypotheses with Mplus 8.5, using DSEM^49^, a useful method for analyzing intensive time-series data as it can control statistical biases via latent variables that focus on analyzing intensive time-series data^50^. Previous studies used DSEM for measuring intensive longitudinal data of rumination relating to state rumination and moods with ESMs^6,22^. We also used DSEM because our aim was to extract dynamic bidirectional associations of how people ruminated; after ruminating (autoregressions), and when they experienced negative moods (cross-lagged effects), from participants’ answer data about their rumination and moods during a day. We set the model as a two-level first-order vector autoregressive model^16^ using Bayesian estimation. The model is presented in Fig. 1.

In the two-level first-order vector autoregressive model, we decomposed the parameters of state rumination and NA as within-person and between-person level variables, as shown in Eq. (1).

1 $$\begin{gathered} RU_{it} = \mu_{RU, i} + RU_{it}^{\left( w \right)} \hfill \\ NA_{it} = \mu_{NA, i} + NA_{it}^{\left( w \right)} \hfill \\ \end{gathered}$$

In ESM, RU is represented as state rumination, NA as negative affect, and μ as the mean of within-person levels. The $RU_{it}^{\left( w \right)}$ and $NA_{it}^{\left( w \right)}$ represent the temporal deviations of individual *I* at occasion *t* from the individual’s means. This decomposition is shown on the left side of Fig. 1. We set the autoregression and cross-lagged paths at the temporal deviations in the within-person level model, which are shown on the right and upper sides of Fig. 1. The within-level deviations $RU_{it}^{\left( w \right)}$ and $NA_{it}^{\left( w \right)}$ on occasion *t* are regressed by the preceding states, such as $RU_{it - 1}^{\left( w \right)}$ and $NA_{it - 1}^{\left( w \right)}$, and thus, autoregression occurs at occasion *t*-1. Moreover, they are regressed by each other, which indicates a cross-lagged effect. These relationships are represented by Eq. (2).

2 $$\begin{gathered} RU_{it}^{\left( w \right)} = \phi_{RR, i} RU_{it - 1}^{\left( w \right)} + \phi_{RN, i} NA_{it - 1}^{\left( w \right)} + \zeta_{RU, t} \hfill \\ NA_{it}^{\left( w \right)} = \phi_{NN, i} NA_{it - 1}^{\left( w \right)} + \phi_{NR, i} RU_{it - 1}^{\left( w \right)} + \zeta_{NA, t} \hfill \\ \end{gathered}$$

The $\phi_{RR, i}$ and $\phi_{NN, i}$ represent autoregression from time *t* − 1, whereas $\phi_{RN, i}$ and $\phi_{NR, i}$ represent the cross-lagged effects from occasions *t* − 1. The $\zeta_{RU, t}$ and $\zeta_{NA, t}$ are residuals that are often said to be innovation^16^, which represent residuals not explained by autoregression or cross-lagged effects. At the between-level, we estimated the effects of each habit and processing mode or covariates on these within-person level coefficients as follows:

3 $$\begin{gathered} \mu_{RU, i} = \gamma_{0. RU} + \gamma_{1. RU} Habit_{i} + \gamma_{2. RU} Processing\;modes_{i} + \gamma_{3. RU} Covariates_{i} + u_{RU, i} \hfill \\ \mu_{NA, i} = \gamma_{0. NA} + \gamma_{1. NA} Habit_{i} + \gamma_{2.NA} Processing\;modes_{i} + \gamma_{3. NA} Covariates_{i} + u_{NA, i} \hfill \\ \phi_{RR, i} = \gamma_{0. RR} + \gamma_{1. RR} Habit_{i} + \gamma_{2. RR} Processing\;modes_{i} + \gamma_{3.RR} Covariates_{i} + u_{RR, i} \hfill \\ \phi_{NN, i} = \gamma_{0. NN} + \gamma_{1. NN} Habit_{i} + \gamma_{2.NN} Processing\;modes_{i} + \gamma_{3.NN} Covariates_{i} + u_{RU, i} \hfill \\ \phi_{NR, i} = \gamma_{0. NR} + \gamma_{1. NR} Habit_{i} + \gamma_{2.NR} Processing\;modes_{i} + \gamma_{3.NR} Covariates_{i} + u_{NR, i} \hfill \\ \phi_{RN, i} = \gamma_{0. RN} + \gamma_{1.RN} Habit_{i} + \gamma_{2.RN} Processing\;modes_{i} + \gamma_{3.RN} Covariates_{i} + u_{RN, i} \hfill \\ \end{gathered}$$

These relationships are shown on the right and lower sides of Fig. 1. To consider the time interval of the ESM, on some days, longer hours (from 11:00 p.m. to 8:00 a.m.) were spent than intervals. To address this, we set the time variable, such that the first response occasion *t* was set as hour 0, and the subsequent occasions from that point were considered elapsed hours. The model was estimated using Bayesian estimation, wherein parameters were set at 10,000 Markov Chain Monte Carlo iterations, and Mplus discards the first half of each chain as being part of the burn-in phase. The results were recorded per 10 iterations, referring to a previous DSEM study^6^. To examine the model in which the data fits best, we compared three models: (1) the simple model, in which there are only ESM variables, not including trait factors and covariates (Model A); (2) the model’s added effects of the habit and processing modes (Model B); and (3) covariates added to Model B (Model C). Using the DIC as a model comparison index, a model with lower scores denotes better information criteria than others^51^. We also used the Bayesian credible interval (95%) to decide whether the coefficients had significant effects (whether it rejected the null hypotheses or not). The Mplus code used in this study is described in the Open Science Framework (please see the data availability statement).

## Data Availability

The data, details of the Japanese version of the HINT scale, and materials are described in the Open Science Framework (https://osf.io/h348t/?view_only=8bdfedd3b71f4341a135df7383e1b227).
